# Deep targeted sequencing in pediatric acute lymphoblastic leukemia unveils distinct mutational patterns between genetic subtypes and novel relapse-associated genes

**DOI:** 10.18632/oncotarget.11773

**Published:** 2016-08-31

**Authors:** C. Mårten Lindqvist, Anders Lundmark, Jessica Nordlund, Eva Freyhult, Diana Ekman, Jonas Carlsson Almlöf, Amanda Raine, Elin Övernäs, Jonas Abrahamsson, Britt-Marie Frost, Dan Grandér, Mats Heyman, Josefine Palle, Erik Forestier, Gudmar Lönnerholm, Eva C. Berglund, Ann-Christine Syvänen

**Affiliations:** ^1^ Department of Medical Sciences, Molecular Medicine and Science for Life Laboratory, Uppsala University, Uppsala, Sweden; ^2^ Cancer Pharmacology and Computational Medicine, Department of Medical Sciences, National Bioinformatics Infrastructure Sweden, Science for Life Laboratory, Uppsala University, Uppsala, Sweden; ^3^ Science for Life Laboratory, Department of Biochemistry and Biophysics, Stockholm University, Stockholm, Sweden; ^4^ Department of Pediatrics, Queen Silvia Children's Hospital, Gothenburg, Sweden; ^5^ Department of Women's and Children's Health, University Children's Hospital, Uppsala, Sweden; ^6^ Department of Oncology-Pathology, Karolinska Institute, Stockholm, Sweden; ^7^ Childhood Cancer Research Unit, Department of Women and Child Health, Astrid Lindgren Children's Hospital, Karolinska University Hospital, Stockholm, Sweden; ^8^ Department of Medical Biosciences, University of Umeå, Umeå, Sweden; ^9^ Nordic Society of Pediatric Hematology and Oncology

**Keywords:** acute lymphoblastic leukemia, targeted next generation sequencing, somatic mutation, relapse, clonal evolution

## Abstract

To characterize the mutational patterns of acute lymphoblastic leukemia (ALL) we performed deep next generation sequencing of 872 cancer genes in 172 diagnostic and 24 relapse samples from 172 pediatric ALL patients. We found an overall greater mutational burden and more driver mutations in T-cell ALL (T-ALL) patients compared to B-cell precursor ALL (BCP-ALL) patients. In addition, the majority of the mutations in T-ALL had occurred in the original leukemic clone, while most of the mutations in BCP-ALL were subclonal. BCP-ALL patients carrying any of the recurrent translocations *ETV6-RUNX1*, *BCR-ABL* or *TCF3-PBX1* harbored few mutations in driver genes compared to other BCP-ALL patients. Specifically in BCP-ALL, we identified *ATRX* as a novel putative driver gene and uncovered an association between somatic mutations in the Notch signaling pathway at ALL diagnosis and increased risk of relapse. Furthermore, we identified *EP300*, *ARID1A* and *SH2B3* as relapse-associated genes. The genes highlighted in our study were frequently involved in epigenetic regulation, associated with germline susceptibility to ALL, and present in minor subclones at diagnosis that became dominant at relapse. We observed a high degree of clonal heterogeneity and evolution between diagnosis and relapse in both BCP-ALL and T-ALL, which could have implications for the treatment efficiency.

## INTRODUCTION

Acute lymphoblastic leukemia (ALL), the most common pediatric cancer, is a genetically heterogeneous disease that arises from the malignant transformation of lymphoid progenitors at different developmental stages. Although impressive improvements in treatment strategies during recent decades have resulted in survival rates now exceeding 85%, relapsed ALL remains a leading cause of cancer-related death in children [1]. Recurrent large-scale chromosomal aberrations in B-cell precursor ALL (BCP-ALL) define genetic subtypes, which are used to support therapeutic decisions and correlate with the clinical outcome. Hyperdiploidy (51–67 chromosomes) and the t(12;21)*ETV6-RUNX1* rearrangement are characteristic for the most common subtypes and are associated with a favorable outcome while MLL rearrangements and hypodiploidy have poor prognosis [2]. About 30% of the pediatric BCP-ALL patients remain uncharacterized by currently used genetic analyses at ALL diagnosis. The T-cell immunophenotype (T-ALL) comprises about 15% of pediatric ALL patients [3].

Next generation sequencing technology has opened up new possibilities to identify cancer mutations. Large-scale sequencing studies of ALL genomes have identified driver mutations that affect lymphoid development and signaling, tumor suppression as well as cell cycle regulation, Ras and tyrosine signaling, cytokine receptors, and epigenetic regulation (reviewed in [4, 5]). Analyses of matched diagnostic and relapse samples have revealed that relapse-acquired mutations are enriched in genes involved in epigenetic regulation, tumor suppression, Ras signaling and drug metabolism [6–10]. It has also been shown that clonal heterogeneity is common in ALL, and that the dominant clone at relapse often evolves from a minor clone present at diagnosis [9].

In the genetic subtypes of ALL studied to date the prevalence of mutations in driver genes differs markedly [4]. With the exception of the finding that patients with MLL-rearrangements harbor very few somatic mutations [11], quantitative comparison between the different subtypes has yet to be performed. To explore the mutational spectrum in the distinct subtypes of ALL and study clonal evolution on the path to relapse, we sequenced 872 cancer genes in a large set of ALL patients, including relapse samples from a subset of the patients. The design of our study, which includes pediatric ALL patients of multiple subtypes, offers a unique opportunity to compare the pathogenesis between the subtypes.

## RESULTS

To comprehensively characterize the patterns of somatic mutations in pediatric ALL, we performed deep next generation sequencing of the exons of 872 cancer genes (Supplementary Table S1) in samples from 337 patients with pediatric ALL. We included all genes in the Cancer Gene Census (http://cancer.sanger.ac.uk/census), and additional genes that have been shown in previous studies to be related to ALL or other types of cancer. Of the 337 patients, 172 were sequenced individually and are referred to as the “diagnostic cohort”. The diagnostic cohort includes 148 BCP-ALL patients and 24 T-ALL patients (Table 1, Supplementary Table S2) from four Swedish pediatric oncology clinics that use harmonized diagnostic criteria and treatment protocols. The BCP-ALL patients comprised 107 patients from eight recurrent cytogenetic subtypes, 19 patients with a normal karyotype and 22 patients with non-recurrent clonal abnormalities as detected by routine cytogenetic analysis at diagnosis. We sequenced a sample collected at diagnosis from all 172 patients in the diagnostic cohort. For 143 of these patients, referred to as the “core cohort”, sequence data from a germline reference sample collected at remission was available [12]. In addition, 24 samples collected at relapse from 19 of the patients were included in the study (Supplementary Table S3). Diagnostic samples from the 165 patients that were not part of the diagnostic cohort were sequenced in pools and are referred to as the “extension cohort”. The extension cohort was used for further investigation of genes predicted as putatively associated with relapse. The different cohorts are illustrated in Supplementary Figure S1.

**Table 1:** Genetic subtypes, clinical outcome and number of driver mutations detected in ALL patients included in the diagnostic cohort (*n* = 172)

Subtype^a^	No. of patients^b^	No. of relapses	No. of other events^c^	Patients with Ras mutations^d^	No. of driver mutations per patient^e^	No. of patients with no driver^f^
T-ALL	24 (20)	2	4	3 (12%)	2.33	5 (21%)
HeH	47 (40)	9	0	29 (62%)	1.26	13 (28%)
t(12;21)	36 (28)	9	1	3 (8%)	0.28	28 (78%)
Other	22 (20)	7	0	11 (50%)	0.68	9 (41%)
Normal	19 (16)	4	1	7 (37%)	0.89	7 (37%)
t(9;22)	8 (5)	3	2	0 (0%)	0.50	4 (50%)
11q23/MLL	4 (4)	2	0	1 (25%)	0.50	3 (75%)
iAMP21	4 (4)	2	0	1 (25%)	0.50	3 (75%)
t(1;19)	4 (4)	1	0	0 (0%)	0.25	3 (75%)
dic(9;20)	3 (2)	1	1	2 (67%)	0.67	1 (33%)
>67chr	1 (0)	0	0	1 (100%)	2.00	0 (0%)

a HeH, high hyperdiploidy (51–67 chromosomes); t(12;21), translocation between the chromosomes (12;21)(p13;q22) *ETV6-RUNX1*; t(9;22), translocation between the chromosomes (9;22)(q11;q34)*BCR-ABL1*; 11q23/MLL, translocation between *MLL* and various other genes; iAMP21, intrachromosomal amplification of chromosome 21; t(1;19), translocation between the chromosomes (1;19)(q23;p13)*TCF3-PBX1*; dic(9;20), dicentric chromosome (9;20)(p13;q11); > 67 chr, > 67 chromosomes; Other, other clonal aberrations; Normal, no genetic aberrations detected and a normal karyotype observed in at least 5 of 25 metaphases. Patients that are not marked T-ALL have the BCP-ALL immunophenotype.

b Within parenthesis is the number of patients that are part of the core cohort of patients with a matched germline sample.

c Other events include death in clinical remission 1 (DCR1), secondary malignancy (SMN), resistant disease and failed induction.

d Number of non-silent mutations in the genes *KRAS*, *NRAS*, *PTPN11* and *FLT3* are listed.

e Mean number of non-silent mutations in 19 predicted driver genes.

f Number of patients that do not have a non-silent mutation in any of the predicted driver genes.

### Somatic mutations at ALL diagnosis

**Figure 1:**
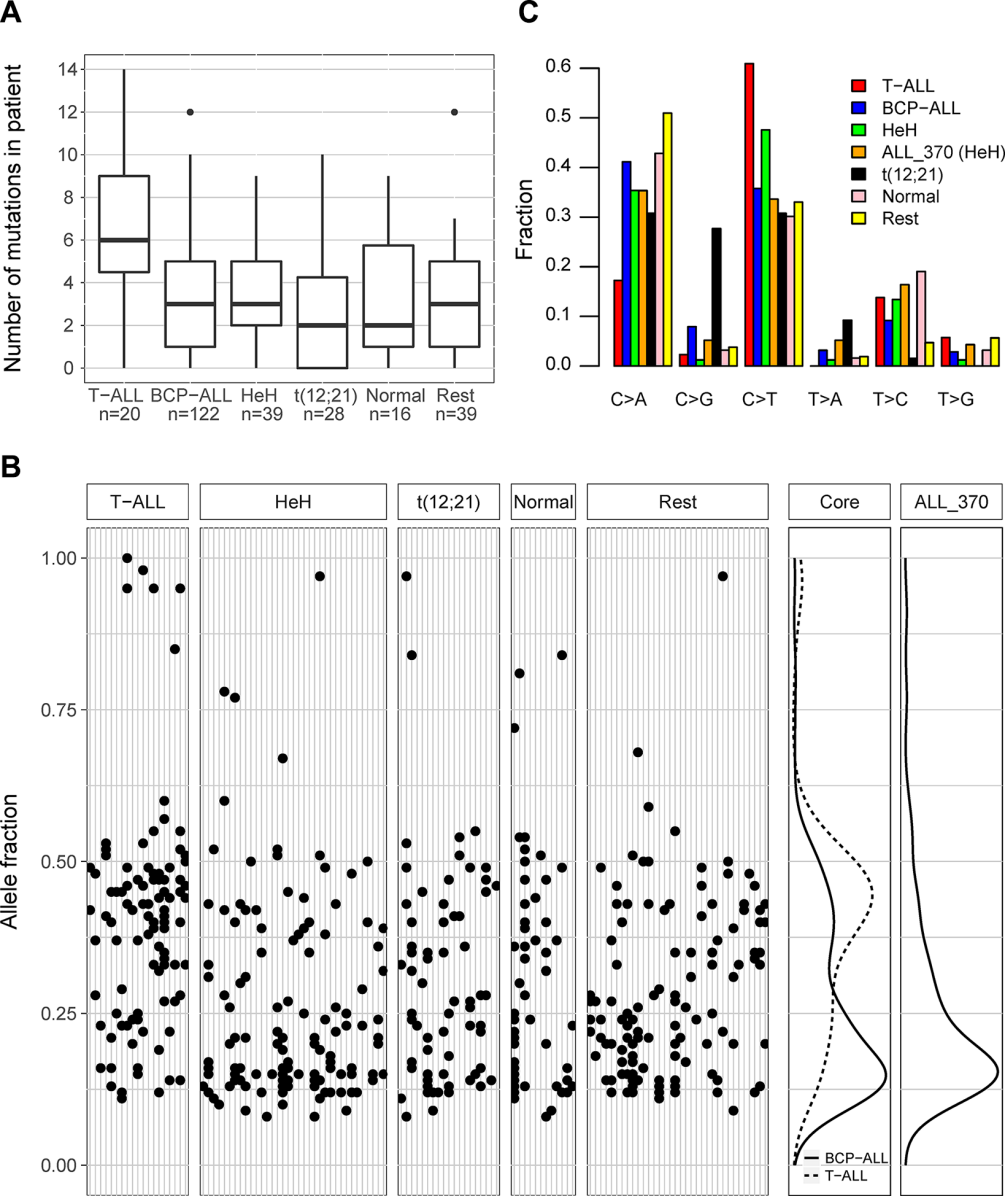
Somatic mutations in acute lymphoblastic leukemia (ALL) (**A**) Boxplots showing the median number of somatic mutations detected at diagnosis in T-ALL, BCP-ALL, and the major genetic subtypes of BCP-ALL. Results from all patients with paired diagnostic and germline samples that constitute the core cohort are shown, excluding the hypermutated patient with the HeH subtype (ALL_370). The number of mutations is shown on the vertical axis and the patient subgroups, with number of individuals in each group, are indicated on the horizontal axis. BCP-ALL patients with non-recurrent genetic aberrations or any of the rare subtypes t(9;22), 11q23/MLL, iAMP21, t(1;19), dic(9;20) are combined into one group denoted “Rest”. For clarity, one 11q23/MLL patient and two with normal karyotype that have more than 14 mutations (*n* = 16, 16, 31) are not shown in the figure although they were included in the analysis. (**B**) Subclonal structure inferred from somatic single nucleotide variants (SNVs) detected at diagnosis in T-ALL and the genetic subtypes of BCP-ALL. The five left-most panels show the allele fraction (AF) of SNVs (*n* = 610) detected in the patients from the core cohort, excluding ALL_370. Each gray vertical line denotes a patient and the black dots represent the mutations identified in each patient. Patients are sorted according to genetic subtype, as indicated above the panels. Mutations with apparently high AF are in most cases located on the X chromosome in males or in mitochondrial DNA. The two panels to the right show density plots of the AF of the SNVs in the core cohort, and ALL_370 (*n* = 117), respectively. In the core cohort, T-ALL and BCP-ALL patients are shown separately. The density peak with a maximum AF close to 0.4 corresponds to mutations presumably present in all leukemic cells, and the second peak with a maximum AF below 0.25 corresponds to subclonal mutations. (**C**) Pattern of single base substitution in patients with T-ALL, BCP-ALL, and the major genetic subtypes of BCP-ALL. The relative fraction of each substitution type is shown for the ALL patients in the core cohort as indicated by the color key.

We detected 973 somatic single nucleotide variants (SNVs), 35 deletions and 34 insertions in the 872 sequenced genes in the diagnostic cohort (*n* = 172) (Supplementary Table S4). In the core cohort (*n* = 143) the average number of somatic mutations detected per patient was 3.9 (range 0–31) (Figure 1A, Supplementary Figure S2), after exclusion of one outlier patient with high hyperdiploid (HeH) ALL (ALL_370) who harbored as many as 120 somatic mutations. We detected significantly more somatic mutations in T-ALL patients compared to BCP-ALL patients (averages of 6.4 and 3.5, *p* = 1.7 *10^−4^, Figure 1A), in line with our previous results from whole genome sequencing of four ALL patients [12]. We found no correlation between number of mutations and age at diagnosis (Supplementary Figure S3), indicating that the difference between the immunophenotype groups was not related to the older age of T-ALL patients (average of 10.1 and 6.9 years in the core cohort). We found no difference in terms of number of mutations between the major BCP-ALL subtypes (Figure 1A). In the 29 BCP-ALL and T-ALL patients that are not part of the core cohort we identified 13.0 (range 3–35) mutations on average (Supplementary Figure S2). The higher number of mutations detected in these patients is expected, since the lack of a matched germline sample will allow rare germline variants to escape filtering. We found no somatic or germline mutations, *e.g.* in DNA repair genes, that could explain the apparent hypermutation in the patient ALL_370. However, we cannot exclude the presence of such mutations since only a subset of all genes was sequenced.

The deep coverage in our sequence data (average 638×) allowed accurate determination of mutant allele fractions that can be used to identify subclones. The allele fractions (AF) in the core cohort were distributed into two peaks, with an estimated boundary between the peaks at AF 0.32 (Figure 1B). A mutation that was present in the original leukemic clone is expected to be present in all leukemic cells at diagnosis, and have an AF slightly below 0.5, depending on the percentage of leukemic cells in the sample (typically 80–95%). Mutations in the peak with low AF are most likely subclonal, *i.e.* they occurred after the establishment of the original clone, and are consequently present only in a subset of the leukemic cells. A larger fraction of the somatic mutations in T-ALL compared to BCP-ALL belong to the peak with high AF (*p* = 3.3 * 10^−10^, Figure 1B, Supplementary Figure S4), while no trend of a difference was observed between the BCP-ALL subtypes (Supplementary Figure S4). Virtually all mutations in the hypermutated BCP-ALL sample (ALL_370) appeared to be subclonal (Figure 1B), despite an estimated percentage of leukemic cells of 90% in this sample.

In the core cohort we observed an overall high abundance of C > T and C > A single-base substitutions across the ALL subtypes (Figure 1C). Based on information on the trinucleotide context of the somatic SNVs (*i.e.*, the nucleotides before and after the SNV), we inferred the predominant mutational signatures in the diagnostic cohort and compared these signatures to previously described signatures [13]. Two such signatures were identified. One of them occurs as the result of an endogenous mutational process that has been observed in all cancer types and is characterized by C > T substitution at methylated cytosines. The second signature is characterized by a high proportion of C > G substitutions at TpCpA or TpCpT motifs (Supplementary Figure S5), was predominantly observed in BCP-ALL patients with the t(12;21) subtype, and is known to be induced by increased activity of the AID/APOBEC family of cytidine deaminases [13]. In comparison to BCP-ALL, the T-ALL patients showed a high fraction of C > T substitutions at ApCpG sequence motifs, both in the core cohort analysed here and in the whole genome sequenced patients in our previous study [12] (Supplementary Figures S6–S7).

### Driver genes

**Figure 2:**
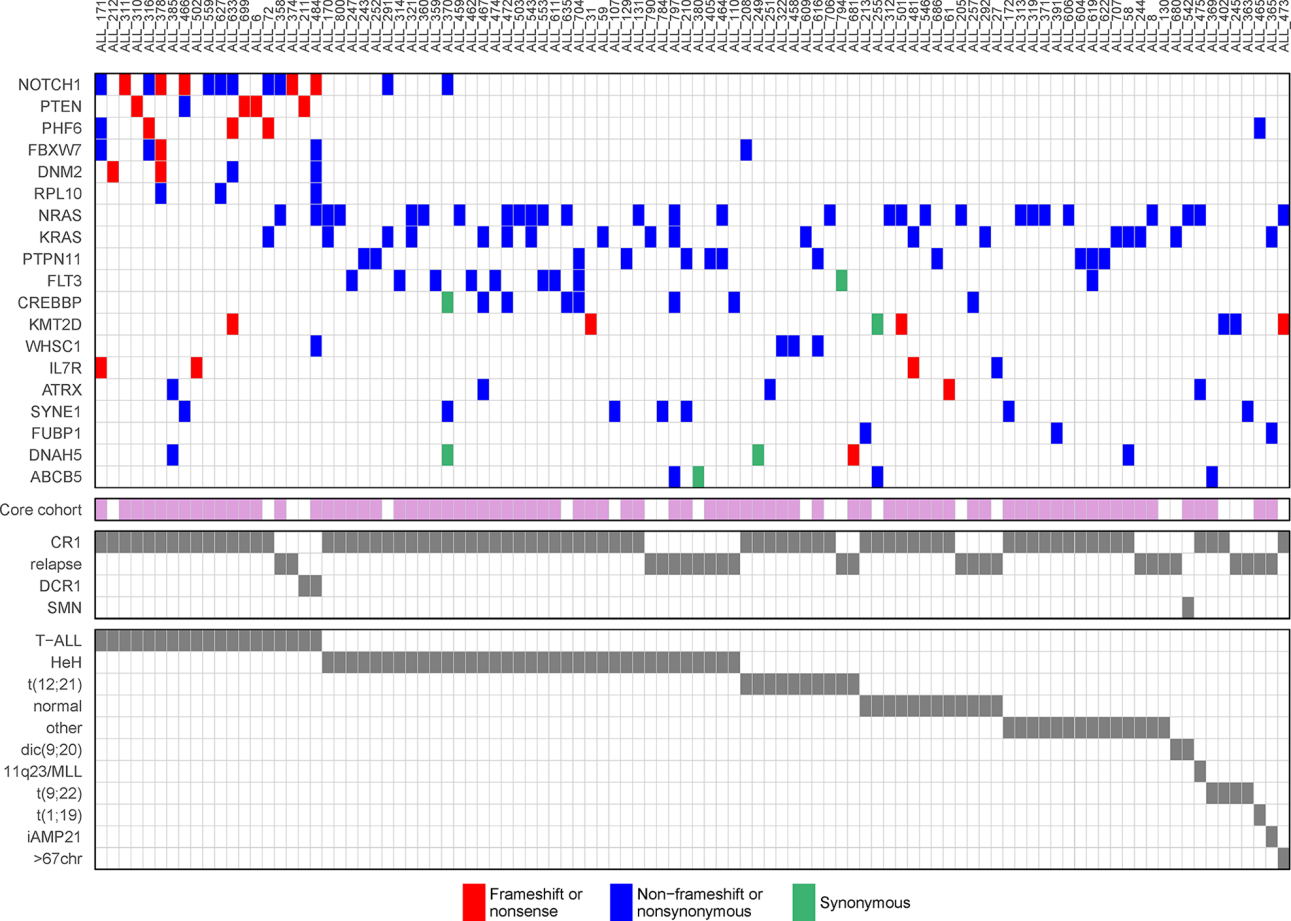
Driver genes in acute lymphoblastic leukemia (ALL) Driver genes identified by computational analysis of somatic mutations detected at ALL diagnosis. In the top panel, each row represents a gene, each column represents an ALL patient, and each colored box indicates a mutation. Patients with at least one mutation in any of the predicted driver genes are shown. For patients with more than one mutation in the same gene, the color is prioritized according to the order shown in the color key in the figure. The violet bars below each column in the top panel indicate patients that are part of the core cohort with a matched germline sample. The clinical outcome and the genetic subtype of each patient are shown by grey bars in the two bottom panels. CR1, clinical remission 1; DCR1, death in CR1; SMN, secondary malignancy.

To identify the genes involved in the development of pediatric ALL we performed computational screening for driver genes based on the somatic mutations in the diagnostic cohort. We identified 19 putative driver genes (Table 2, Figure 2, Supplementary Figure S8) using a combination of three bioinformatics tools that are based on the assumptions that driver genes have more mutations than expected by chance, more mutations with a high functional impact, or tight clustering of mutations within a gene. Twelve of the 19 genes are well known driver genes in ALL from previous studies, which demonstrates the robustness of our results. We also replicate the finding of *RPL10* as a T-ALL driver, which has only been reported once before [14]. *KMT2D* was previously found by us in the same cohort of ALL patients [12], and the *ATRX*, *SYNE1, FUBP1*, *DNAH5*, and *ABCB5* genes, which were all identified in BCP-ALL, have not previously been described as driver genes for ALL.

**Table 2:** Driver genes identified in ALL patients included in the diagnostic cohort (*n* = 172)

Gene	Gene description	Cytoband	GEX (FPKM)^a^	ALL subtype^b^	ALL association
NOTCH1	notch 1	9q34.3	7.8	T-ALL	T-ALL [21]
PTEN	phosphatase and tensin homolog	10q23	152	T-ALL	T-ALL [55]
PHF6	PHD finger protein 6	Xq26	13.1	T-ALL	T-ALL [56]
FBXW7	F-box and WD repeat domain containing 7	4q31.23	23.7	T-ALL	T-ALL [57]
DNM2	dynamin 2	19p13.2	11.2	T-ALL	T-ALL [27]
RPL10	ribosomal protein L10	Xq28	339.1	T-ALL	T-ALL [14]
NRAS	neuroblastoma RAS viral oncogene homolog	1p13.2	31	BCP-ALL, HeH, normal, T-ALL	ALL [58]
KRAS	KRAS proto-oncogene, GTPase	12p12.1	10.9	BCP-ALL, HeH	ALL [59]
PTPN11	protein tyrosine phosphatase, non-receptor type 11	12q24.1	48.6	BCP-ALL, HeH	BCP-ALL [60]
FLT3	fms related tyrosine kinase 3	13q12	61.3	BCP-ALL, HeH	HeH [61], 11q/MLL [62]
CREBBP	CREB binding protein	16p13.3	9	BCP-ALL, HeH	BCP-ALL [6]
KMT2D	lysine methyltransferase 2D	12q13.12	15.4	BCP-ALL	ALL [12]
WHSC1	Wolf-Hirschhorn syndrome candidate 1	4p16.3	48.2	BCP-ALL, t(12,21)	t(12;21) [63]
IL7R	interleukin 7 receptor	5p13	39.9	BCP-ALL, normal, T-ALL	BCP-ALL [64], T-ALL [65]
ATRX	alpha thalassemia/mental retardation syndrome X-linked	Xq21.1	41.1	BCP-ALL	
SYNE1	spectrin repeat containing nuclear envelope protein 1	6q25.2	12.7	BCP-ALL, HeH	
FUBP1	far upstream element binding protein 1	1p31.1	102.4	BCP-ALL	
DNAH5	dynein axonemal heavy chain 5	5p15.2	0	BCP-ALL	
ABCB5	ATP binding cassette subfamily B member 5	7p14	0.1	BCP-ALL	

a The shown expression value represents the mean of 18 T-ALL samples (for the genes identified as drivers only in T-ALL) or 27 BCP-ALL samples (for the remaining genes). FPKM, fragments per kilobase of transcripts per million mapped reads.

b The ALL immunophenotype(s) and subtype(s) in which the gene was identified as a driver. BCP-ALL signifies that the gene was identified as driver in the set of all BCP-ALL samples.

**Figure 3:**
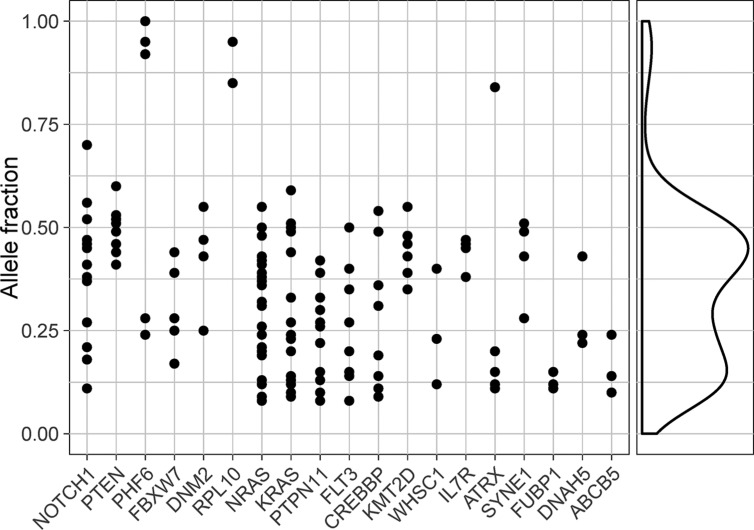
Allele fractions of somatic mutations in driver genes Allele fractions of all non-silent somatic mutations (*n* = 167) detected in predicted driver genes at ALL diagnosis. Allele fractions are plotted on the vertical axis and gene names are shown on the horizontal axis. To the right of the large panel is a density plot which includes all mutations shown in the figure.

Because solely the identification of a gene by a computational tool does not imply that the gene is a driver gene, we analyzed the five novel putative driver genes in more detail. Each of the 14 previously described ALL driver genes was expressed on the RNA level and harbored mutations that were present in the original leukemic clone (Table 2, Figure 3). Thus it is plausible that novel driver genes would also show the same pattern in terms of gene expression and clonality, a criterion only fulfilled by *ATRX* and *SYNE1* (Table 2, Figure 3). Computational predictions by PolyPhen-2 and SIFT suggested that virtually all *ATRX* and *SYNE1* mutations could be deleterious (Supplementary Table S5). However, inspection of the protein sequence and structure indicated that none of the *SYNE1* mutations have a strong effect on the protein (Supplementary Table S5). In addition, *SYNE1* is one of the largest genes in the human genome and its function appears unrelated to hematopoiesis and cancer. Thus, *SYNE1* might not be a true driver gene in ALL. In contrast, several of the *ATRX* mutations are likely to have an effect on the protein (Supplementary Table S5), and this gene might represent a novel ALL driver gene.

We observed major differences in terms of driver genes between T-ALL and BCP-ALL patients. The most prevalent driver mutations were in *NOTCH1* in T-ALL and in the Ras signaling pathway (*NRAS*, *KRAS*, *PTPN11* and *FLT3*) in BCP-ALL (Figure 2, Supplementary Figure S8). We found a non-frameshift deletion in the known ALL driver gene *FLT3* in five HeH patients, which to our knowledge has not previously been reported as a recurrent mutation in ALL. T-ALL patients harbored on average more mutations in putative driver genes than BCP-ALL patients, and were less likely to lack a driver mutation (p = 9.7 * 10^−5^, Table 1, Supplementary Figure S9). Significant differences in number of putative driver mutations were also observed between the BCP-ALL subtypes, with most driver mutations in patients with HeH and few mutations in patients with the recurrent translocations *ETV6-RUNX1*, *BCR-ABL,* or *TCF3-PBX1* (*p* = 1.9 * 10^−5^, Table 1, Supplementary Figures S9). The driver mutations in the Ras signaling pathway displayed a similar pattern, with few mutations in patients with recurrent translocations (Supplementary Figure S10). The only gene that was identified as a driver in the t(12;21) BCP-ALL patients was *WHSC1* (*NSD2*) (Supplementary Figure S8). A total of 76 patients (44%) in the diagnostic cohort did not harbor any non-silent mutation in the 19 predicted driver genes (Table 1). In contrast to a previous study in chronic lymphoblastic leukemia, where patients with more driver mutations displayed a lower overall survival [15], we found no correlation between the number of putative driver mutations and clinical outcome (Supplementary Figure S11).

### Genes and pathways associated with relapse

**Figure 4:**
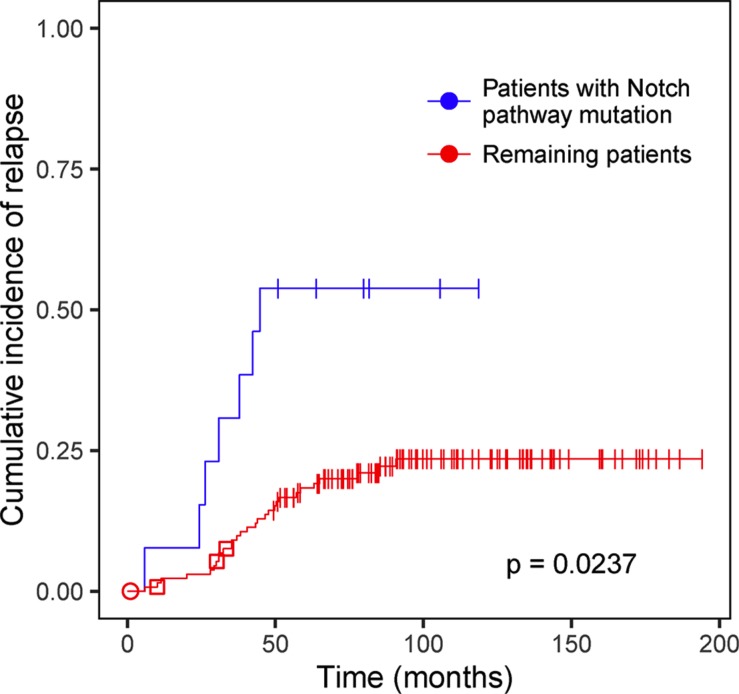
Survival analysis of acute lymphoblastic leukemia (ALL) patients with and without mutations in the Notch signaling pathway Cumulative incidence of relapse in BCP-ALL patients in the diagnostic cohort with mutations in the Notch signaling pathway. The blue line represents patients that have at least one non-silent mutation in the Notch signaling pathway and the red line represents the remaining BCP-ALL patients. The Bonferroni-corrected Gray's test *p*-value is shown. The symbols shown on the cumulative incidence curves represent the clinical outcome of patients that did not relapse. Vertical bars indicate clinical remission 1 (CR1), circles indicate resistant disease, and squares indicate secondary malignancy.

To identify genes and pathways that are associated with relapse in ALL, we performed survival analysis using Gray's test based on the somatic mutations detected in the diagnostic cohort. We analyzed all genes that harbored non-silent mutations in at least five patients in a specific patient subgroup (*n* = 0–10 genes per group). No single gene was found to be significantly associated with relapse. In a separate analysis, mutations were assigned to pathways according to the Kyoto Encyclopedia of Genes and Genomes (KEGG). All pathways that were mutated in at least five patients in a subgroup (*n* = 10–60 pathways per group) were included in the analysis. We found an increased risk of relapse for BCP-ALL patients with a mutation in the Notch signaling pathway (*p* = 0.0237, Figure 4). Mutations in genes in the Notch pathway in patients that relapsed included nonsynonymous SNVs (nsSNVs) in *CREBBP* (*n* = 3), *EP300* (*n* = 1), *MAML2* (*n* = 1), *HDAC2* (*n* = 1), *NOTCH2* (*n* = 1) and *DTX1* (*n* = 1), all of which are expressed in BCP-ALL (Supplementary Table S5). Computational predictions and functional evidence suggest that each of these patients harbored a damaging mutation in the Notch pathway at diagnosis (Supplementary Table S5). We also identified Notch pathway mutations that were called only at relapse in *CREBBP* (*n* = 3), which is a known relapse-associated gene in ALL [6], *EP300* (*n* = 2), *NOTCH1* (*n* = 1) and *NOTCH2* (*n* = 1). *EP300* is a paralog to *CREBBP* which has not yet been shown as relapse-associated in ALL, and the recurrent mutations in this gene are thus highly interesting. Investigation of the extension cohort identified one additional non-silent mutation in *EP300*, and the patient that harbored this mutation also relapsed (Table 3, Supplementary Table S5).

**Table 3:** Somatic mutations in genes putatively associated with ALL relapse

Gene	Protein change	Patient^a^	Subtype^b^	SIFT^c^	PP2^d^	Origin^e^	AF^f^	GEX (FPKM)^g^	Functional evidence^h^
AFF3	p.I941V	ALL_260	t(9;22)	T	B	Dia (E)	NA	87	Small change in amino acid properties *
AFF3	p.E811K	ALL_784	HeH	T	D	Dia (D)	0.46	87	Disordered part of the protein that folds upon binding. Could affect protein-protein interaction [66]
AFF3	NA	ALL_128	other	NA	NA	Rel 2	0.16	87	Splice site SNV *
ARID1A	p.T626P	ALL_217	T-ALL	D	D	Dia (E)	NA	20.8	Close to DNA-binding where the proline's ability to bend the backbone is likely to affect the binding [67] *
ARID1A	p.R893X	ALL_358	T-ALL	NA	NA	Rel 1	0.30	20.8	Premature stop codon that removes the majority of the protein *
EBF1	p.V440L	ALL_54	HeH	T	B	Dia (E)	NA	131.1	Small change in amino acid properties *
EBF1	p.N100fs	ALL_27	normal	NA	NA	Rel 1	0.41	131.1	Frameshift that removes the majority of the protein *
EBF1	p.N100RN	ALL_27	normal	NA	NA	Rel 1	0.41	131.1	In DNA-binding domain but far away from the DNA binding site [68, 69] *
EBF1	p.N100FGN	ALL_168	t(9;22)	NA	NA	Dia (D)	0.40	131.1	In DNA-binding domain but far away from the DNA binding site [68, 69] *
EP300	p.N700K	ALL_365	iAMP21	T	D	Dia (D)	0.24	10.7	Medium change in amino acid properties *
EP300	p.Q734R	ALL_821	other	T	D	Dia (E)	NA	10.7	Small change in amino acid properties *
EP300	p.A794T	ALL_244	other	T	D	Rel 1	0.49	10.7	Medium change in amino acid properties *
EP300	p.S225SN	ALL_5	11q23/MLL	NA	NA	Rel 2	0.27	10.7	Located close to a region involved in various protein-protein interactions *

a All patients relapsed except ALL_168 who suffered from a secondary malignancy.

b For explanation of the subtypes, see legend to Table 1.

c SIFT predictions: D, damaging; T, tolerated.

d PolyPhen-2 (PP2) predictions: D, probably damaging; P, possibly damaging; B, benign.

e Indicates at which disease state (Dia, diagnosis; Rel 1, first relapse; Rel 2, second relapse) and in which cohort (D, diagnostic; E, extension) the mutation was first called.

f The mutations identified in the extension cohort have no allele fraction (AF) since they were called in pools of samples.

g The shown expression value represents the mean of 18 T-ALL samples (for *ARID1A*) or 27 BCP-ALL samples (for the remaining genes). FPKM, fragments per kilobase of transcripts per million mapped reads.

h A star indicates that no structural information was available for this region of the protein.

Additional information about these mutations is available in Supplementary Table S5.

A mutation that was called at relapse but not at diagnosis has either occurred after the diagnostic sample was collected or it was present at diagnosis in a subclone too small to be identified by standard variant calling methods (see the next section on clonal evolution). Since in both cases, the mutation is present in a much larger proportion of leukemic cells at relapse compared to diagnosis and could have been involved in driving the relapse, we refer to these mutations collectively as “relapse-gained”. In addition to the *CREBBP* and *EP300* mutations described above, recurrent non-silent relapse-gained mutations were detected in *NOTCH1* (*n* = 3), *KRAS* (*n* = 2) and *NT5C2* (*n* = 2), all of which are known in ALL, as well as *SYNE1* (*n* = 2) and *MUC5B* (*n* = 2). Both relapse-gained mutations in *SYNE1* appear benign (Supplementary Table S5), and *MUC5B* is not expressed in ALL. By joint analysis of relapse-gained and diagnostic mutations, including the extension cohort, we found that BCP-ALL patients with *EBF1* or *AFF3* mutations and T-ALL patients with *ARID1A* mutations relapsed frequently. Six patients, of which five relapsed and one experienced a secondary malignancy, harbored mutations in *EBF1* or *AFF3* (Table 3, Supplementary Table S5). However, computational prediction and structural information indicate that several of these mutations have no major effect on protein function (Table 3, Supplementary Table S5). Thus, although these genes are highly relevant in the context of BCP-ALL, their putative association with relapse needs to be further investigated in larger studies. Out of four T-ALL patients in the diagnostic and extension cohorts who relapsed, two harbored mutations in *ARID1A.* The mutations included a nonsense SNV and a nsSNV that introduced a proline at a position where it is likely to disrupt DNA binding (Table 3, Supplementary Table S5). Thus both mutations in *ARID1A* are predicted to have a strong functional impact on the encoded protein.

### Clonal evolution between diagnosis and relapse

**Figure 5:**
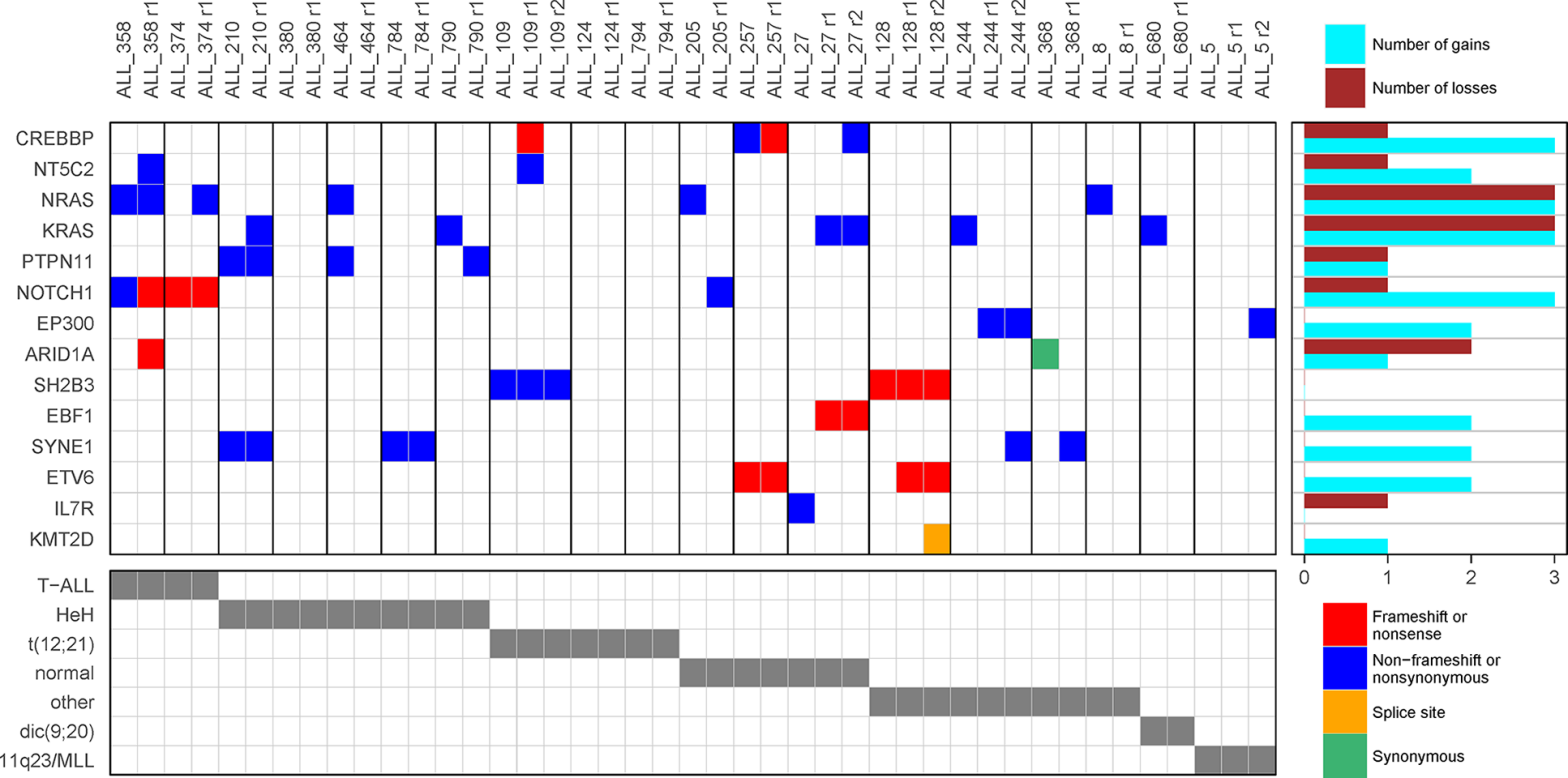
Gain and loss of driver mutations at relapse of acute lymphoblastic leukemia (ALL) Putative driver mutations detected at diagnosis and relapse in 19 ALL patients from whom relapse samples were sequenced. Each column represents an ALL sample with vertical black lines to mark separation between patients. Samples from the same patient are sorted chronologically (diagnosis, first relapse, second relapse). In the main panel, each row represents a gene and each colored box indicates a mutation. For samples with more than one mutation in the same gene, the color is prioritized according to the order shown in the legend. The numbers of gained and lost mutations are summarized to the right of the main panel for each gene. The genetic subtype of each patient is shown in grey in the lower panel.

**Figure 6:**
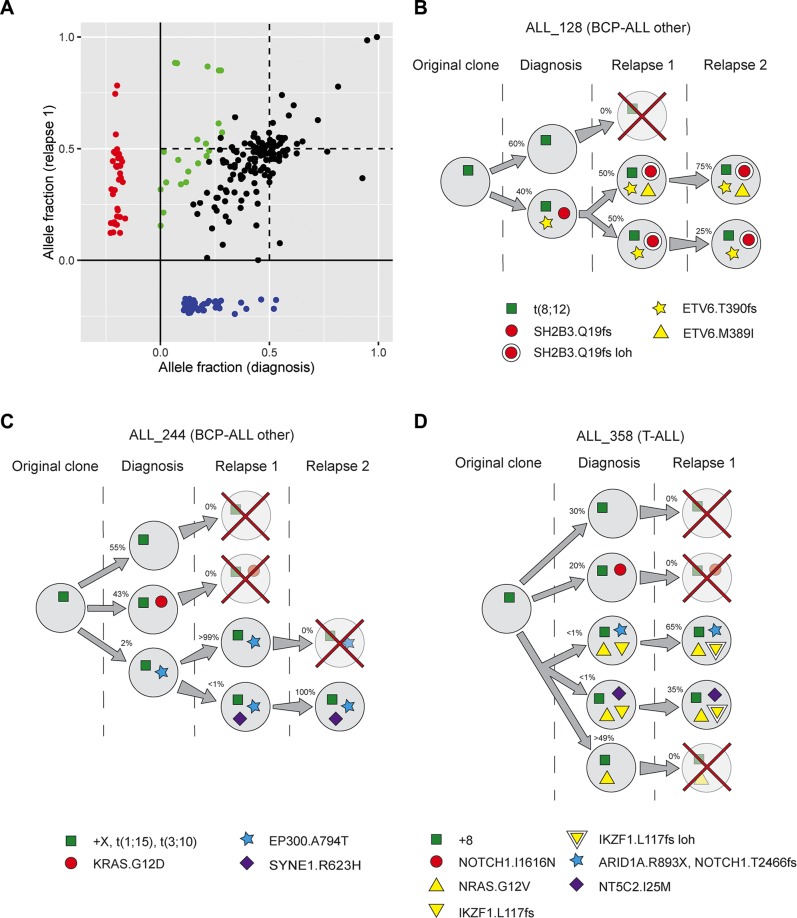
Clonal evolution of acute lymphoblastic leukemia (ALL) cells (**A**) Allele fractions of somatic mutations detected in the 19 patients from whom relapse samples were sequenced. Mutations detected at both diagnosis and relapse are shown in the large square, and those with indications of being part of rising clones are highlighted in green. The rectangular areas at the bottom and the left show mutations only detected at diagnosis (blue) and relapse (red), respectively. (**B–D**) Examples of clonal evolution patterns in the BCPALL patients ALL_128 and ALL_244 with non-recurrent clonal aberrations, and in T-ALL patient ALL_358. Each circle represents an inferred subclone, marked with the estimated percentage of leukemic cells in this clone. The green squares represent aberrations detected by cytogenetic analysis. The most important mutations in each clone are shown. A red cross indicates that the clone is inferred to have been eradicated by therapy. A double circle or triangle indicates loss of heterozygosity (loh). The figures illustrate examples of clonal evolution, and do not necessarily represent the only possible evolutionary path in these patients. (B) ALL_128 harbored a subclone with frameshift indels in *SH2B3* and *ETV6* at diagnosis, which expanded at the first relapse. In addition, an allele fraction of 0.85 for the *SH2B3* indel suggests that the wild type allele of this gene had been lost, and a novel nonsynonymous SNV in *ETV6* which appears to be on the other allele than the indel was identified. All mutations remained at the second relapse. (C) Two subclones were inferred at diagnosis in ALL_244, one with a *KRAS* mutation which was lost at relapse and one with an *EP300* mutation which became dominant at the first relapse. At the first relapse we detected a minor clone with a *SYNE1* mutation, which became dominant at the second relapse. (D) In ALL_358 we inferred as many as four distinct subclones at diagnosis. One subclone harbored a *NOTCH1* mutation and was lost at relapse, and the other three shared an *NRAS* mutation. The two minor *NRAS* clones, which expanded at relapse, also harbored *ARID1A* and *NT5C2* mutations, respectively. At relapse, the *ARID1A* clone acquired a novel *NOTCH1* mutation.

In the 19 patients from whom paired diagnosis-relapse samples were sequenced we identified 313 somatic mutations, including 69 mutations that were called only at diagnosis and 95 that were relapse-gained (Supplementary Tables S4, S6 and S7). We detected subclonal mutations at diagnosis and gain and/or loss of mutations at relapse in each of the 19 patients. Among the ALL driver genes, gain and loss of mutations were particularly common in the genes from the Ras pathway, with seven out of nine non-silent diagnostic Ras mutations lost at relapse and six relapse-gained mutations (Figure 5). Since we sequenced to a high coverage, we were able to determine whether mutations that were only called at relapse were in fact present in a minor subclone already at diagnosis by examining single reads that supported the variant. We found that 20 of the 95 relapse-gained mutations were detectable at low level (AF < 2%) at diagnosis, and six additional mutations that were called only at the second relapse were detectable at low level at the first relapse (Figure 6A, Supplementary Figure S12). These results indicate that relapse clones are often derived from minor subclones that are present already at diagnosis and survive therapy. In contrast, only six of the 69 mutations called only at diagnosis were detectable at relapse, which indicates that it is a rare event that the prevalence of a clone is diminished without being eradicated.

While the allele fractions of mutations that were called at both diagnosis and first relapse were in most cases relatively similar (Figure 6A), nine patients showed evidence of an expanding clone. Such rising clones are particularly interesting because they could harbor mutations that confer resistance to therapy. We have selected two BCP-ALL patients with non-recurrent clonal aberrations (ALL_128 and ALL_244) and one T-ALL patient (ALL_358) to illustrate the patterns of rising clones (Figure 6B–6D). In ALL_128 we observed simultaneous expansion of a clone harboring loss-of-function mutations in *SH2B3* and *ETV6* and loss of the wild type alleles of these genes which resulted in mutations present in a large proportion of leukemic cells (Figure 6B). We inferred two distinct rising clones in both ALL_244 (Figure 6C) and ALL_358 (Figure 6D). Two of these clones harbored mutations in *EP300* or *ARID1A*, which were both identified as putatively associated with relapse (see previous section on genes associated with relapse), and one clone harbored a mutation in the previously known relapse-associated gene *NT5C2* [7, 16].

## DISCUSSION

In the current study, we describe somatic mutations detected by deep targeted sequencing in a large cohort of pediatric ALL patients comprising different genetic subtypes. In light of the recent demonstration that the strategies used for both sequencing and data analysis can greatly influence the results of somatic variant calling [17], our study design is particularly useful for comparison of the mutational landscapes between immunophenotypes and subtypes of ALL. We found several differences between the T-ALL and BCP-ALL groups. These distinctive differences include an overall larger number of mutations and a larger number of mutations in driver genes in T-ALL. The high sequence depth of our study allowed accurate determination of allele fractions, which enabled us to observe that a higher proportion of the somatic mutations in T-ALL than in BCP-ALL were present already in the original leukemic clone. A possible explanation for this is that T-ALL generally has a faster and more explosive course than BCP-ALL, thus there has been less time to collect additional mutations since the establishment of the ancestral leukemic clone.

A striking difference between the BCP-ALL subtypes is that the patients carrying the recurrent fusion genes *ETV6-RUNX1*, *BCR-ABL* or *TCF3-PBX1* harbored exceptionally few mutations in known driver genes compared to the other BCP-ALL patients. This finding may indicate that the fusion genes themselves act as strong drivers, so that few or no additional driver mutations are required to induce ALL. Our finding that the APOBEC signature of nucleotide substitution is only observed in patients with the *ETV6-RUNX1* fusion gene and the previous demonstration of frequent RAG-mediated recombination in this subtype [18] suggests that different molecular mechanisms are active in patients with the *ETV6-RUNX1* fusion gene than in the other ALL subtypes. Thus it would be interesting to study the mutational patterns in larger cohorts of patients with other recurrent fusion genes. Patients of the HeH subtype stood out as having many driver mutations, in particular in the Ras pathway. In contrast to a previous study [19], we found no indications of mutual exclusivity between Ras mutations in the HeH patients.

We found that BCP-ALL patients with mutations in the Notch signaling pathway relapsed frequently. The Notch pathway is a well-known genetic determinant in T-ALL and has been implicated in several other cancers [20]. While the majority of somatic mutations in *NOTCH1* in T-ALL are activating and result in aberrant continued signaling [21], a tumor suppressor role of the Notch pathway has been shown in acute myeloid leukemia (AML) [22, 23]. Although the majority of the Notch pathway mutations detected in our study are predicted to have a functional effect on the protein, there is no evidence suggesting that they would be activating. Thus, it seems reasonable to assume that the mutations in *CREBBP*, *EP300* and *MAML*, which all encode transcriptional co-activators, could result in reduced Notch signaling. In contrast, *HDAC2* and *DTX1* are negative regulators of Notch signaling, and mutations in these genes are more likely to result in increased signaling. Given the complex role of the Notch pathway in hematological malignancies, it is not unlikely that different types of disturbances in Notch signaling could be involved in the progress of ALL.

Our results also highlighted the *ATRX*, *EP300*, *ARID1A* and *SH2B3* genes as novel putative ALL drivers, associated with relapse, or being present in rising clones at relapse. All genes except *ARID1A* were identified in the BCP-ALL patient group. *ATRX* is a known driver gene in other cancers than ALL [24]. It encodes a chromatin remodelling protein whose activity affects genomic stability and heterochromatin structure. Although the mechanisms by which loss of ATRX leads to cancer progression are unclear, we find that all ATRX mutations detected in our study are predicted to be deleterious.

*EP300* is a known target of translocation in ALL [25], and has recently been reported to be recurrently mutated in adult T-ALL [26] and early T-cell precursor T-ALL [27], but not yet in BCP-ALL. *EP300* is also a known driver gene in other types of cancer [28], and germline variants in *EP300* have been shown to confer inherited risk of pediatric ALL in a Hispanic population [29]. There is evidence for somatic mutations in the paralogous gene *CREBBP* that result in impaired histone acetylation and transcription of *CREBBP* target genes, including glucocorticoid-receptor-responsive genes, which may influence response to therapy and the likelihood of relapse in ALL [6]. In light of this evidence, it will be crucial for future studies to investigate the effect of *EP300* mutations in ALL.

*ARID1A* is recurrently mutated in several cancer types, including lymphomas [30]. The encoded protein contains a well conserved DNA-binding domain which is also present in several other proteins with related functions. Interestingly, a putatively relapse-associated mutation in *ARID4B* has been identified in pediatric T-ALL [31], and *ARID5B* has been associated with germline susceptibility to pediatric ALL [32]. ARID1A is a chromatin remodeler that appears to act as a tumor suppressor, and decreased expression of *ARID1A* is associated with poor prognosis in gastric cancer [33] and hepatocellular carcinoma [34]. Previous studies have shown that 97% of somatic mutations in *ARID1A* are inactivating [30], in line with the predictions of the mutations detected in our study. While 30% of *ARID1A* mutations in the literature affect both alleles, 73% of the cases lack protein expression, implicating that haploinsufficiency for *ARID1A* may be enough to promote tumor formation [35, 36]. Thus, although the mutations identified in *ARID1A* in our study were most likely heterozygous, these mutations could be involved in ALL progression.

Homozygous germline mutations in *SH2B3* have previously been found in familial ALL, and somatic loss of both *SH2B3* alleles has been described in a few BCP-ALL patients [37]. *SH2B3* encodes a lymphocyte adaptor protein that plays a key role in hematopoiesis and has been suggested to be a tumor suppressor [37]. Our study is to our knowledge the first to report a heterozygous loss-of-function mutation in *SH2B3* at diagnosis that becomes homozygous at relapse, and suggests that the homozygous loss of *SH2B3* could have been a driving factor for relapse in this patient.

Our analysis of allele fractions at diagnosis and relapse in the deep sequencing data revealed that many apparently relapse-gained mutations were present in minor clones at diagnosis. The targeted approach used here enabled detection of small leukemic subclones with one experiment. Our approach is time- and cost-efficient compared to exome or whole genome sequencing followed by targeted deep-sequencing of sites with somatic mutations, but a smaller proportion of all somatic mutations will be identified. For example, a recent study of clonal evolution in ALL using exome sequencing identified a median of 18 SNVs and indels at diagnosis [9] in comparison to a median of 3 in our study. While a larger set of mutations enables more robust clustering into subclones, we expect that our approach identified the majority of the putative driver mutations and their evolution between diagnosis and relapse.

In summary, by targeted sequencing of cancer genes in ALL samples collected at diagnosis and relapse, we identified distinctive differences in the mutational landscape between the immunophenotypes and genetic subtypes of ALL, discovered *ATRX* as a novel putative driver gene in ALL and identified *EP300*, *ARID1A* and *SH2B3* as relapse-associated genes. An interesting observation in our study was that germline variants in *EP300*, *SH2B3* and *ARID5B* have previously been associated with ALL susceptibility. Although the heritability in ALL is low, genome-wide association studies have identified germline mutations in several genes that are also affected by somatic mutations, including *IKZF1, ARID5B, CEBPE* and *CDKN2A* [32, 38]. Likewise, studies of familial ALL have revealed inherited mutations in *PAX5* [39] and *ETV6* [40], both of which are also targets of somatic mutation and chromosomal translocation in ALL. Another interesting feature is that all four genes mentioned above are involved in epigenetic regulation. Somatic mutations in epigenetic regulators play a role in many cancer types, including T-ALL [41, 42]. In BCP-ALL, it has been shown that mutations in epigenetic regulators are gained during relapse [8, 9]. Our results are in line with these previous findings, with mutations in *EP300*, *ARID1A* and *SH2B3* being present in rising clones at relapse. We observed a high degree of clonal heterogeneity and evolution between diagnosis and relapse, which could have implications for the treatment efficiency.

## MATERIALS AND METHODS

### Patient samples

The pediatric ALL patients analyzed in this study were diagnosed and treated at Swedish pediatric oncology centers in Uppsala, Umeå, Stockholm and Gothenburg, according to the Nordic Society for Pediatric Haematology and Oncology (NOPHO) protocols [43]. ALL diagnosis was established by analysis of leukemic cells with respect to morphology, immunophenotype, and cytogenetics. Immunophenotype (BCP-ALL or T-ALL) was defined according to the European Group for the Immunological Characterization of Leukemias. Gene fusions were screened for by fluorescence *in situ* hybridization (FISH) or reverse transcriptase polymerase chain reaction (RT-PCR). Karyotypes were assigned according to the International System for Human Cytogenetic Nomenclature [44]. DNA and RNA were extracted as described previously [12]. Targeted sequencing of 172 samples collected at diagnosis (“diagnostic cohort”) and 24 samples collected at relapse from 19 patients was performed (Table 1, Supplementary Tables S2 and S3). The percent of leukemic cells was at least 80 in the diagnostic samples (median 90%). For 163 of the 172 patients, a matched germline sample collected during first clinical remission (CR1) was sequenced in pools. We have previously shown, using germline SNPs that were unique for each patient, that 143 of the 163 remission samples were adequately represented in the pools [12]. In addition, a cohort of 165 samples collected at diagnosis (“extension cohort”) which were sequenced in pools (Lindqvist *et al*, manuscript in preparation) and used to further investigate putative relapse-associated genes (Supplementary Table S8). An in-house RNA-sequencing dataset containing 27 BCP-ALL samples and 18 T-ALL samples (Nordlund *et al*, unpublished data) was used to determine gene expression levels in ALL cells. The study was approved by the Regional Ethical Review Board in Uppsala, Sweden. The study was conducted according to the guidelines of the Declaration of Helsinki, and all patients and/or guardians provided informed consent.

### Target capture and next generation sequencing

The exons of 872 genes related to cancer were selected for resequencing (Supplementary Table S1). Target capture was performed using 200 ng of DNA and reagents from a HaloPlex Target Enrichment kit (Agilent), according to the Automation Protocol Version D.3. The 172 samples in the diagnostic cohort and the 24 relapse samples were enriched individually. Remission samples were enriched in pools of ten samples. The 165 samples in the extension cohort were enriched in pools of ten samples without a unique individual barcode for each sample. However, each sample was present in two pools using a design that enabled assignment of rare somatic mutations to their carrier (Lindqvist *et al*, manuscript in preparation). In addition, 84 samples from healthy Swedish blood donors were enriched in pools of 21 samples and used for filtering purposes. Paired-end sequencing with 100 bp reads was performed on a HiSeq2000/2500 system (Illumina). The average sequence depth per sample in the target region was 638× for ALL samples enriched individually (diagnostic cohort and relapse samples), 529× for ALL samples in pools (extension cohort), 162× for remission samples and 133× for Swedish blood donors.

### Analysis of sequence data

Variant calling in the diagnostic cohort and relapse samples was performed using FreeBayes (http://arxiv.org/abs/1207.3907) for SNVs and the GATK HaplotypeCaller [45] for indels. Variants were filtered based on sequence coverage, quality scores and germline variants, and annotated against the Ensembl database as described previously [12]. Putative driver genes were identified using MutSigCV [46], Oncodrive-fm [47] and OncodriveCLUST [48] as described previously [12], except that OncodriveCLUST version 0.4.1 was used. For this analysis, the 172 patients in the diagnostic cohort were divided into five subsets: 1) T-ALL, 2) BCP-ALL, 3) BCP-ALL with HeH, 4) BCP-ALL with the translocation t(12;21), and 5) BCP-ALL with a normal karyotype. A gene was considered as a putative driver if it was predicted as a driver in at least one of the patient subgroups and harbored at least three non-silent mutations. RNA sequence data was generated and analyzed as described previously [12]. All somatic variants called in the diagnostic cohort or in the relapse samples are listed in Supplementary Tables S4, S6 and S7.

### Analysis of mutational signatures

Signatures of mutational processes were determined using the R package somatic signatures [49]. The analysis was performed on SNVs from the core cohort and from the four previously whole genome sequenced patients [12]. All recurrent SNVs (*i.e.* SNVs present in more than one patient) were excluded. The samples in the core cohort were divided into the same five groups as described above, except that ALL_370 was analyzed individually. The four whole genome sequenced patients were also analyzed individually. The trinucleotide context of all SNVs was determined using function mutation context and reference sequence from package BSgenome.Hsapiens.UCSC.hg19. Motif frequencies of the groups from the core cohort were then corrected according to differences in motif occurrence in the target region relative to the whole genome using the functions kmerFrequency and normalize motifs. Decomposition of the motif matrix to three signatures was done using non-negative matrix factorization with the function identify signatures. The similarity between mutational signatures identified in our data and previously validated mutational signatures available at http://cancer.sanger.ac.uk/cosmic/signatures was assessed using hierarchical clustering with the agglomeration method complete on a Euclidean distance matrix of the scaled signature matrix.

### Analysis of protein structures and protein sequences

Protein sequence motifs and functional sites were predicted based on ProRule [50] and ProSite [51]. The nsSNVs located in protein domains with determined NMR or X-Ray structure deposited in the Protein Data Bank [52] or homologous structure was visually inspected to predict the effect of nsSNVs on the protein.

### Survival analysis

Survival analysis with Gray's test [53], taking the competing risks death in clinical remission 1 (DCR1), resistant disease and secondary malignancy (SMN) into account, was performed for genes and pathways, assessing the association between clinical outcome (relapse) and mutation status. Only non-silent mutations were included, and samples from infants (< 1 year) were excluded from the analysis (*n* = 3). Patients were analyzed in the following subgroups: 1) the complete data set, 2) T-ALL samples, 3) BCP-ALL samples, 4) BCP-ALL samples stratified by risk group (high, intermediate and standard risk), 5) BCP-ALL with HeH, 6) BCP-ALL with t(12;21), and 7) BCP-ALL with normal karyotype. To avoid spurious results caused by small patient groups, only genes and pathways that were mutated in at least five patients were considered. For analysis at the gene level, we compared patients with and without a somatic mutation in each gene. For analysis at the pathway level, we compared patients with and without a somatic mutation in any of the genes belonging to the pathway. Genes were assigned to pathways according to the Kyoto Encyclopedia of Genes and Genomes (http://www.genome.jp/kegg). Multiple testing correction was performed using the Bonferroni method [54], correcting for the number of genes or pathways that harbored non-silent mutations in at least five patients in the specific patient subgroup.

### Analysis of relapse-associated genes and clonal evolution

A gene was defined as putatively relapse-associated if it fulfilled at least one of the following two criteria: 1) we detected at least two non-silent relapse-gained mutations (*i.e.*, mutations called only at relapse) in the gene, or 2) we detected at least one relapse-gained mutation in the gene, and additional mutations at diagnosis in patients in the diagnostic and extension cohorts that relapsed. For genes in the second category, we did not allow non-silent mutations at diagnosis in patients of the same immunophenotype that remained in CR1.

To determine the allele fraction of somatic mutations at time points where they had not been called by FreeBayes, we used the GENOTYPE_GIVEN_ALLELES mode of GATK HaplotypeCaller [45]. For analysis of clonal evolution between diagnosis and relapse, somatic mutations were clustered manually, based on in which of the samples they were detectable and the allele fraction at these different time points. Although the clustering is somewhat arbitrary due to the low number of mutations, it still reflects the dynamics of gained and lost mutations during leukemic progression. A mutation is considered to be part of a rising clone if the allele fraction at relapse is at least twice the allele fraction at diagnosis.

### Statistical analysis

The number of somatic mutations in different patient subgroups was compared with Wilcoxon rank sum test. Correlation between number of mutations and age at diagnosis was performed using Spearman's rank correlation. Comparisons between the fraction of mutations in the original clone, and the number of driver mutations in different patient subgroups was performed with the chi-square test. The association between number of driver mutations and clinical outcome was tested with a Poisson regression model, excluding 10 BCP-ALL patients and three T-ALL patients with a shorter follow-up time than five years.

## SUPPLEMENTARY MATERIALS FIGURES AND TABLES




