# Supplementary material for: Deep targeted sequencing in pediatric acute lymphoblastic leukemia unveils distinct mutational patterns between genetic subtypes and novel relapse-associated genes
Source: Oncotarget. 2016 Aug 31;7(39):64071–88. doi: 10.18632/oncotarget.11773 (PMC5325426; doi:10.18632/oncotarget.11773)
Supplement: Supplementary file 1 [file oncotarget-07-64071-s001.pdf]

Deep targeted sequencing in pediatric acute lymphoblastic leukemia unveils distinct mutational patterns between genetic subtypes and novel relapse-associated genes

Supplementary Materials

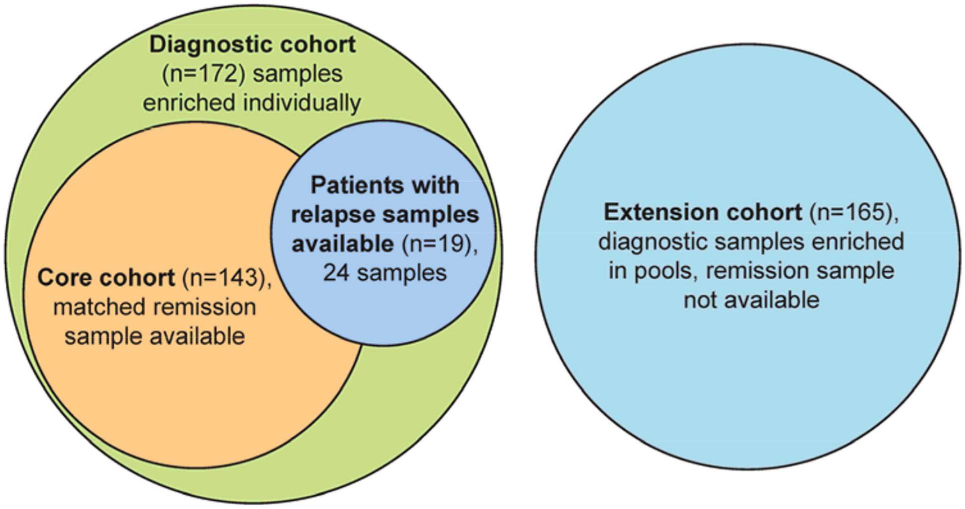

Supplementary Figure S1: Patient cohorts. Schematic illustration of the patients and samples analyzed in the present study. The diagnostic cohort from which the samples were subjected individually to enrichment using the HaloPlex system forms the basis of the study. For the majority of the patients in the diagnostic cohort (*n* = 163), a matched germline (remission) sample was available and was enriched in pools of ten samples. The core cohort (*n* =143) contains the patients for whom a matched germline sample was sequenced and a good representation of this sample in the pool had been established by targeted analysis of patient-specific SNPs. For 19 of the patients in the diagnostic cohort, one or two relapse samples were sequenced (*n* =24 relapse samples in total). A matched germline sample was available for nine of these 19 patients. Ten patients in the diagnostic cohort are neither part of the core cohort nor had a relapse sample available. The extension cohort contains samples sequenced in pools for whom no matched germline sample was available. Each sample was sequenced in two pools, using a design that allowed rare mutations to be assigned to their carrier (Lindqvist et al, manuscript in preparation). The extension cohort was used exclusively for further investigation of genes identified as putatively associated with relapse.

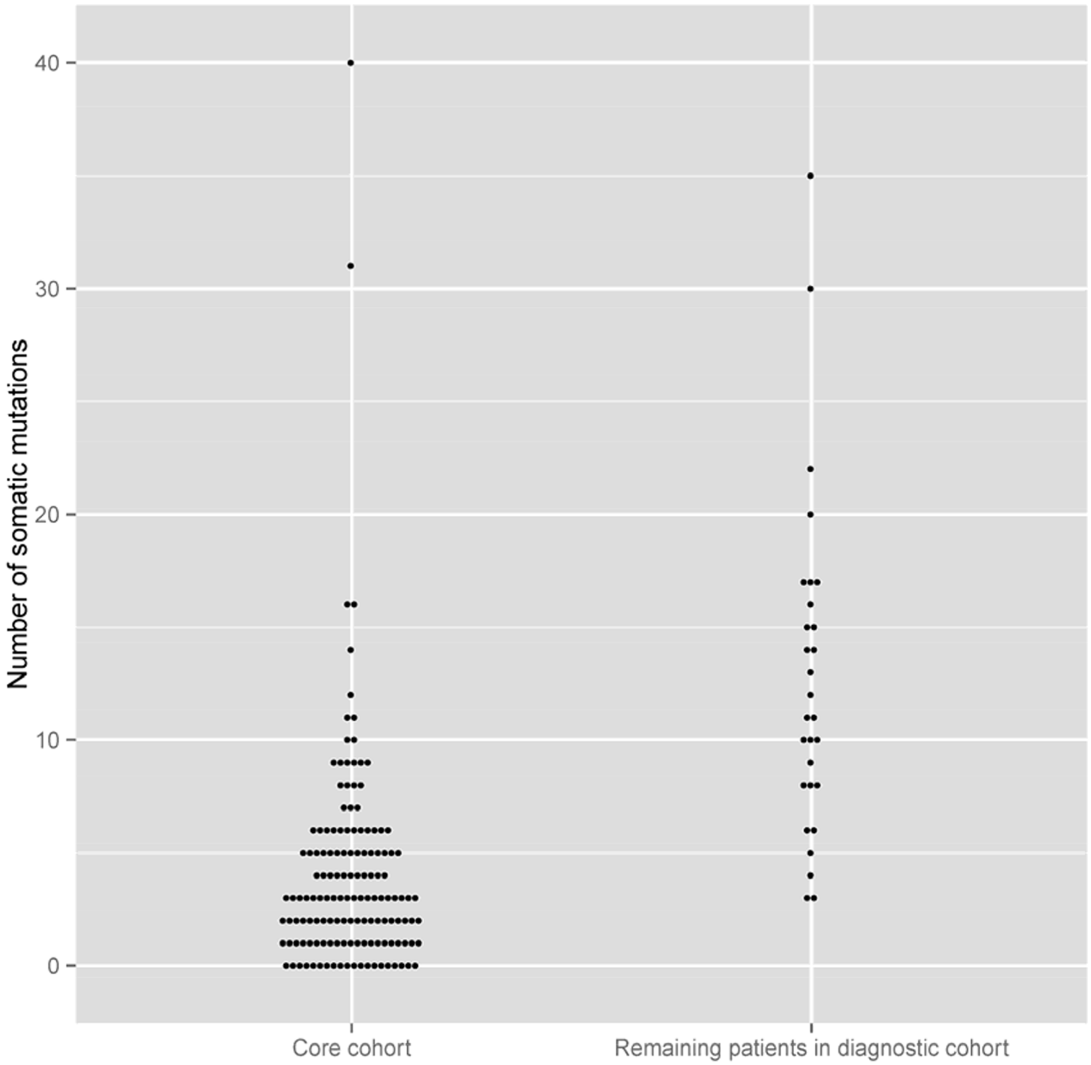

Supplementary Figure S2: Number of somatic mutations in patients with and without a matched germline sample. The dots represent the number of somatic mutations detected in each patient in the diagnostic cohort. Mutations detected in patients that belong to the core cohort with a matched germline sample (*n* =143) are shown on the left and patients that do not have a germline reference sample (*n* =29) are shown on the right. Patient ALL_370, who is part of the core cohort and harbored 120 somatic mutations, is not shown.

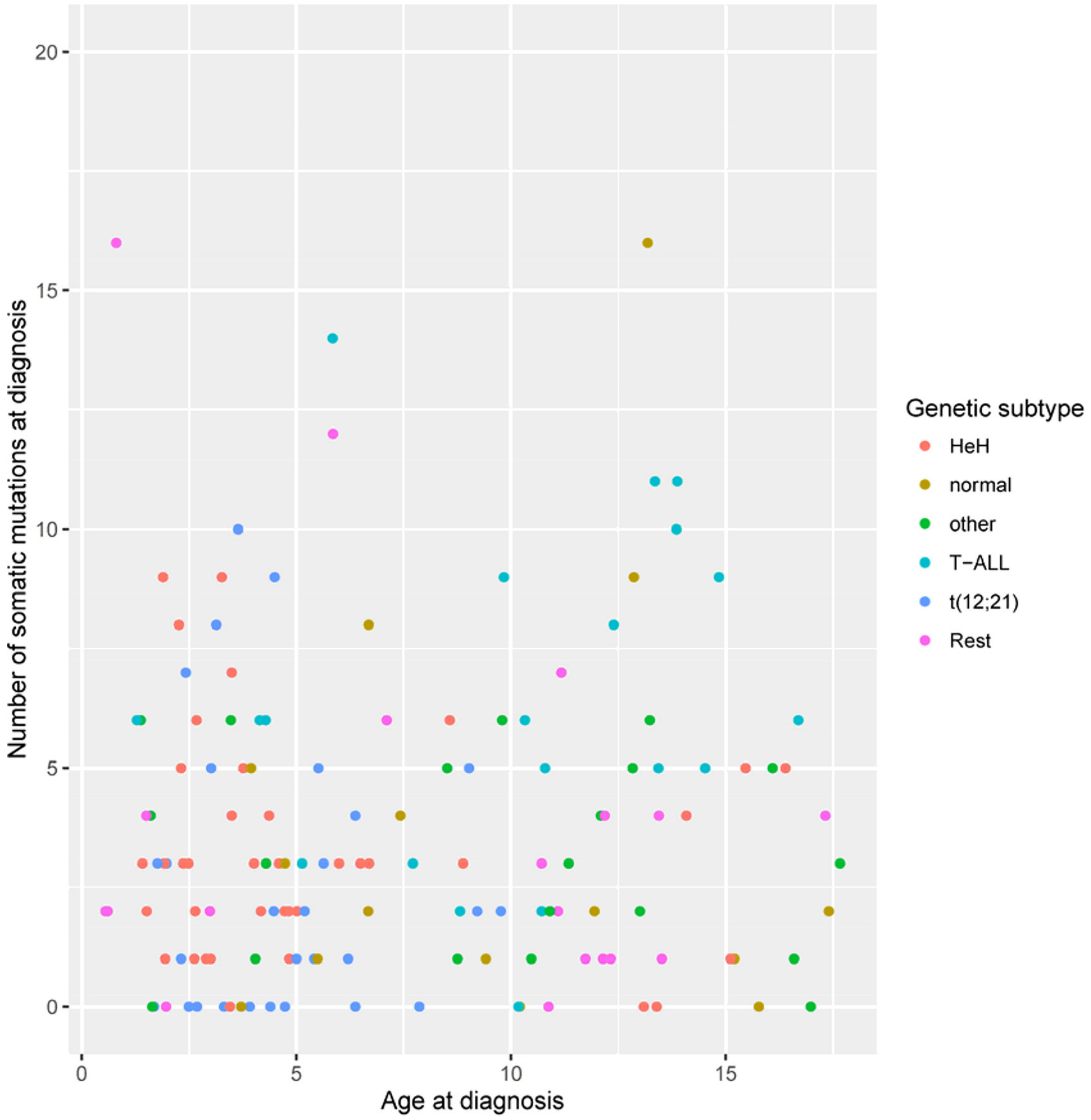

Supplementary Figure S3: Number of detected somatic mutations in ALL patients of different age at diagnosis. The horizontal axis shows the age of each patient at diagnosis and the vertical axis shows the number of somatic mutations detected at diagnosis. Each dot represents one patient, color-coded according to genetic subtype as shown in the legend. Only patients in the core cohort are shown. No correlation was observed between the number of mutations and age.

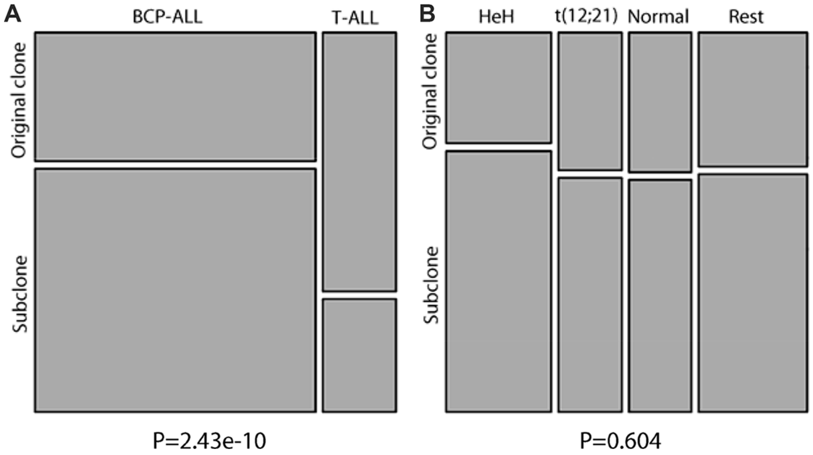

Supplementary Figure S4: Distribution of single nucleotide variants (SNVs) in the original clone and subclones in different patient subgroups. The figures show the proportion of SNVs that most likely were present in the original clone, and those that are likely to be subclonal. SNVs detected at diagnosis in patients in the core cohort, excluding ALL_370, are shown. (**A**) A significantly larger proportion of the SNVs belonged to the original clone in T-ALL patients compared to BCP-ALL patients. (**B**) No difference was observed between the major BCP-ALL subtypes.

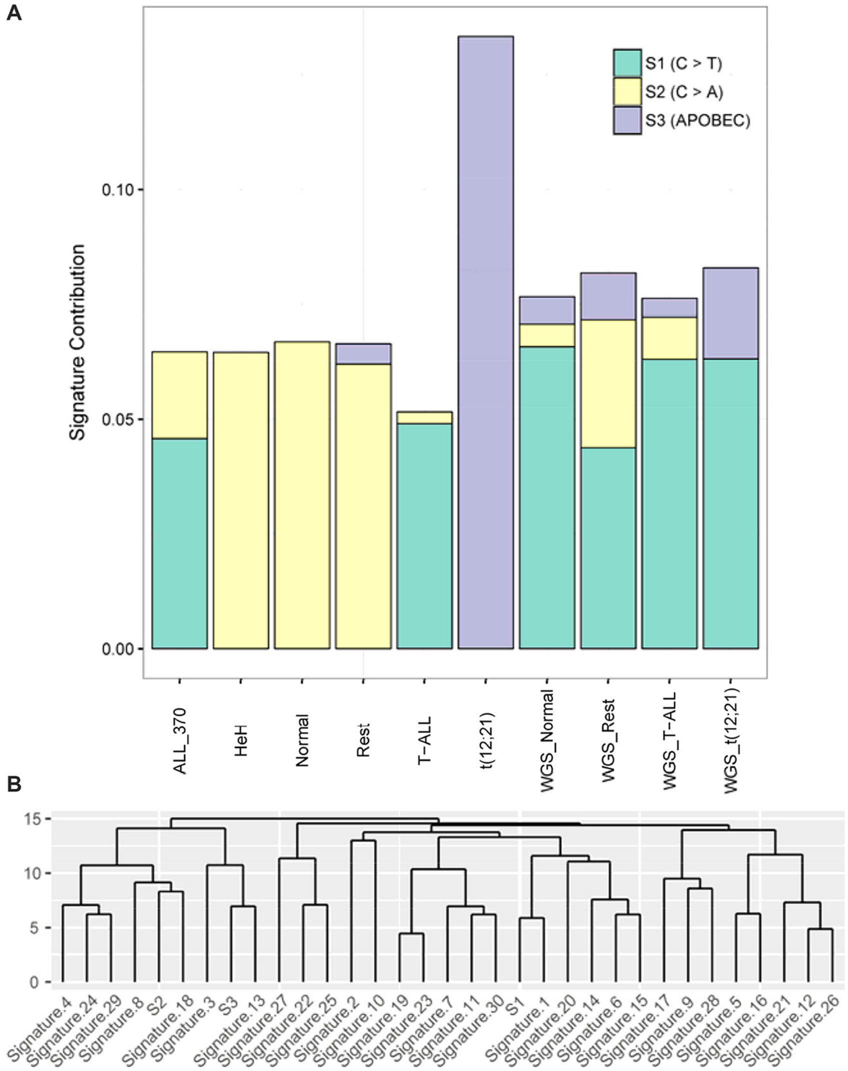

Supplementary Figure S5: Mutational signatures detected in the major ALL subtypes and four whole genome sequenced patients. (**A**) Mutational signatures detected in subtypes of the ALL patients in the core cohort and four whole genome sequenced patients. Whole genome sequenced samples are denoted by "WGS" followed by genetic subtype of each patient. The signatures were inferred from somatic mutations detected in our cohort, and subsequently compared to previously described signatures. Signature S1 represents endogenous C > T substitutions at methylated cytosines, and S3 is the APOBEC signature with a high proportion of C > G substitutions at TpCpA or TpCpT motifs. S2 is characterized by many C > A mutations in a pattern that shows no strong similarity with previously described signatures. (**B**) Hierarchical clustering of the mutational signatures detected in our data (S1–S3) together with 30 previously described signatures. Description of the known signatures, which were identified by analysis of somatic single nucleotide variants in > 11,000 samples across 40 types of human cancer, is available at http://cancer.sanger.ac.uk/cosmic/signatures.

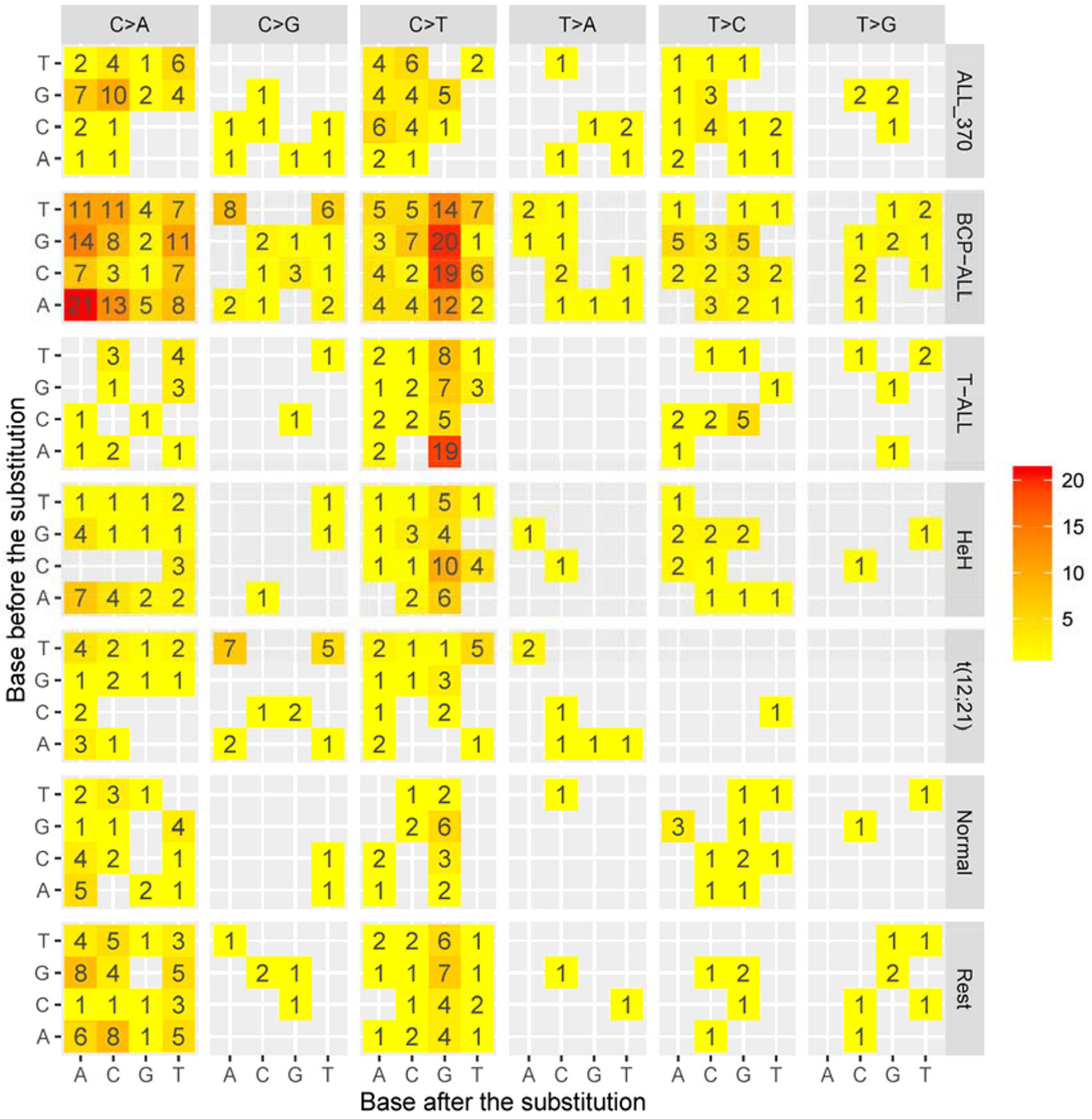

Supplementary Figure S6: Patterns of single nucleotide substitutions in different patient subgroups. Single nucleotide variants (SNVs) are divided into 96 categories, based on the actual nucleotide substitution detected at diagnosis in the core cohort and the two nucleotides flanking the substitution. The nucleotide substitution is shown on top, with *e.g.* "C > A" indicating a C-to-A mutation. The base before the substitution is shown along the left vertical axis, and the base after the substitution is shown along the bottom horizontal axis. The subtypes of the patients are shown on the right vertical axis. The color code and the number at each node in the matrix indicate the number of observed somatic SNVs in each category of trinucleotides. Patient ALL_370, with 117 SNVs, is excluded from the HeH group and shown separately.

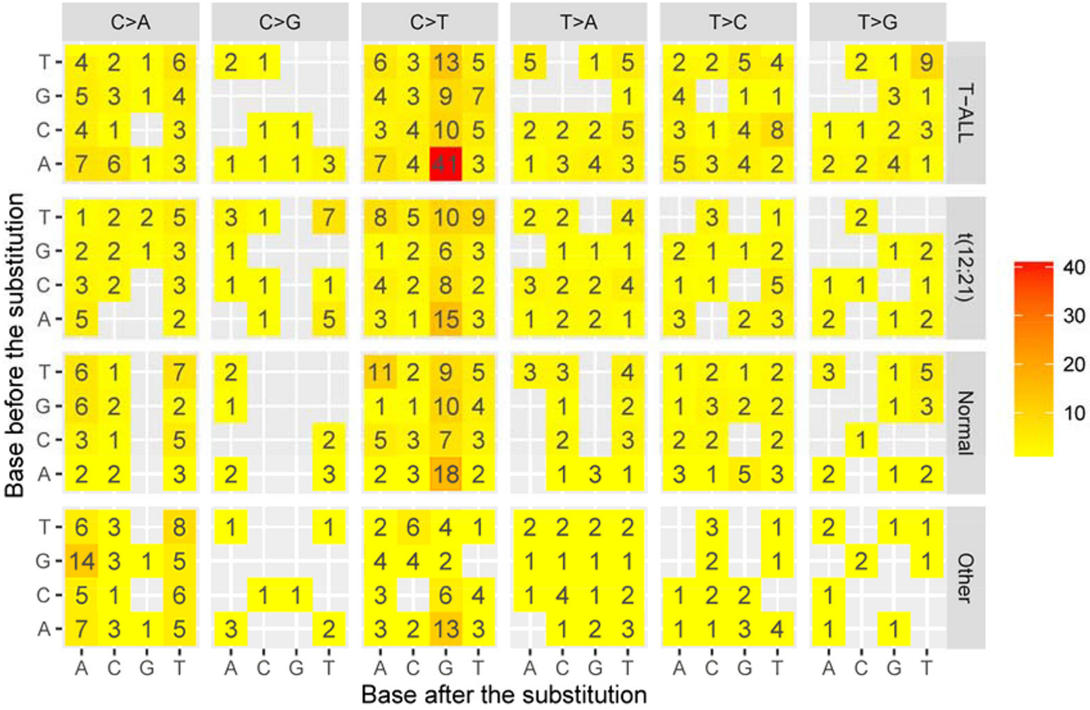

Supplementary Figure S7: Pattern of single nucleotide substitutions in four whole genome sequenced patients. Single nucleotide variants (SNVs) are divided into 96 categories, based on the actual nucleotide substitution and the two nucleotides flanking the substitution. The nucleotide substitution is shown on top, with *e.g.* "C > A" indicating a C-to-A mutation. The base before the substitution is shown along the left vertical axis, and the base after the substitution is shown along the bottom horizontal axis. The subtypes of the patients are shown on the right vertical axis. The color code and the number at each node in the matrix indicate the number of observed somatic SNVs in each category of trinucleotides. All SNVs (n = 907) detected in the four whole genome sequenced patients are shown.

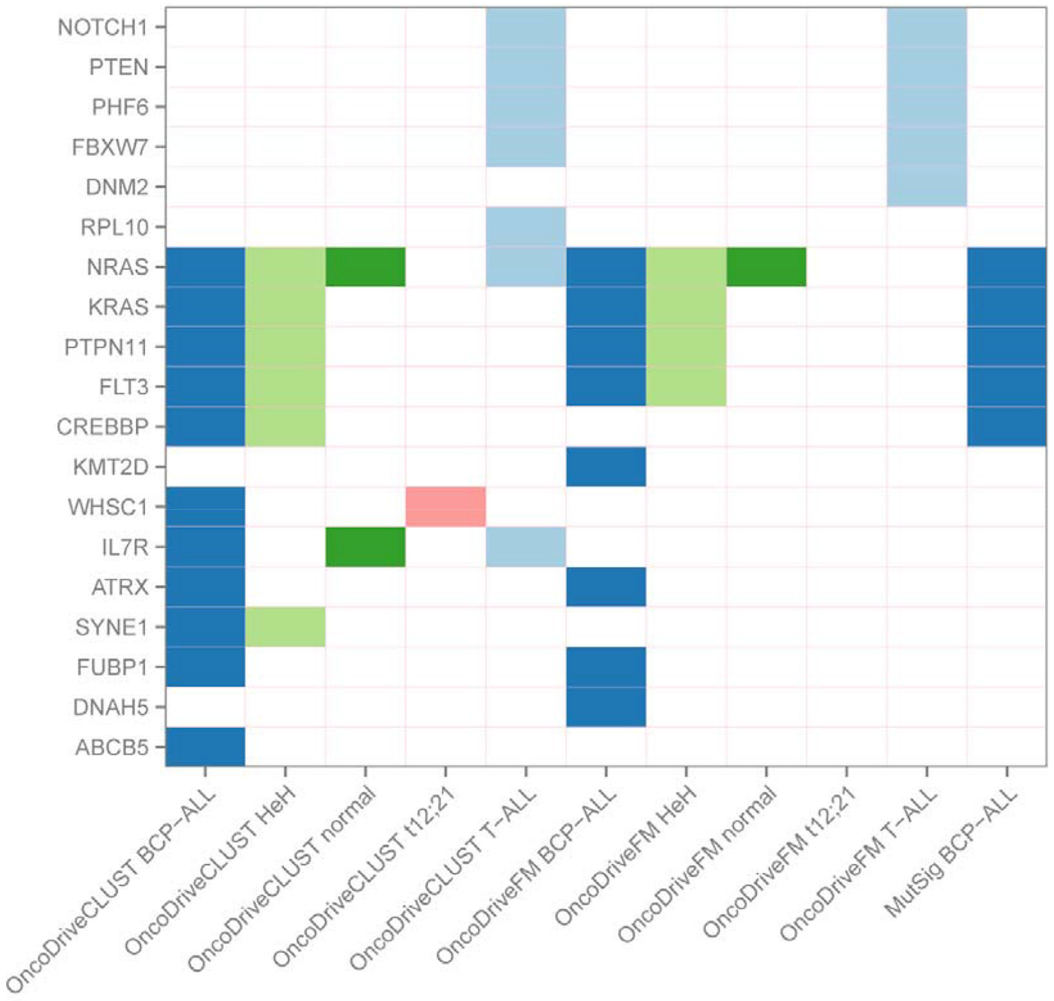

Supplementary Figure S8: Predicted driver genes in different patient subgroups. Three computational tools were used for driver gene prediction: OncodriveClust, Oncodrive-fm and MutSigCV. Each column represents a combination of analysis software and patient subgroup, and each row represents one gene. A colored box indicates that the gene on that row was identified as a putative driver gene in the patient subgroup indicated on the horizontal axis by the analysis software also indicated on the horizontal axis. Analysis with MutSigCV required a larger number of samples than the Oncodrive programs, and could therefore only be applied for analysis of the complete set of BCP-ALL samples.

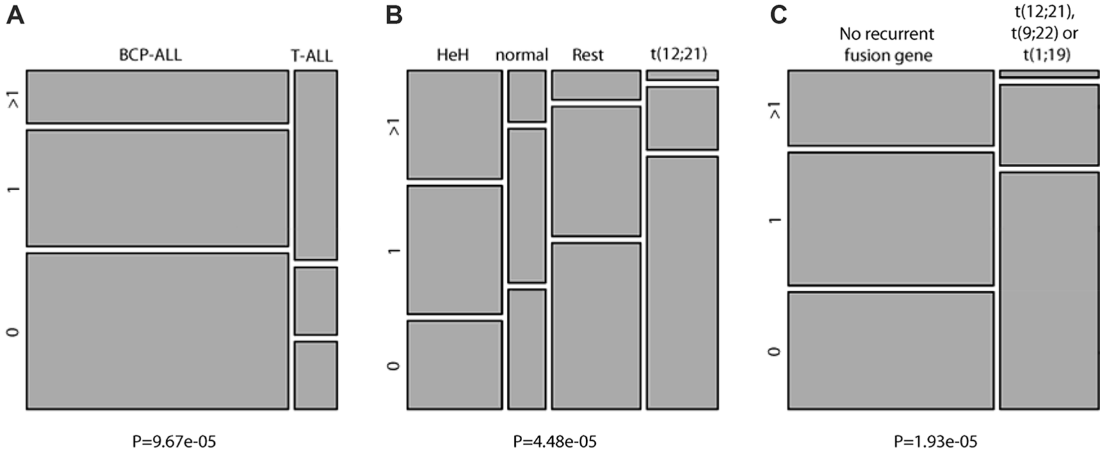

Supplementary Figure S9: Frequency of driver mutations in different patient subgroups. The panels show the proportion of patients with 0, 1 and >1 putative driver mutation. Non-silent somatic mutations in any of the 19 predicted driver genes detected at diagnosis in any patient in the diagnostic cohort are shown. (**A**) T-ALL patients had significantly more putative driver mutations than BCP-ALL patients. (**B**) Significant differences in number of putative driver mutations were observed between the major BCP-ALL subgroups, with the largest number of in HeH patients and the lowest number in t(12;21) patients. (**C**) BCP-ALL patients with any of the recurrent fusion genes *ETV6-RUNX1*, *BCR-ABL* or *TCF3-PBX1* had fewer putative driver mutations than the remaining BCP-ALL patients.

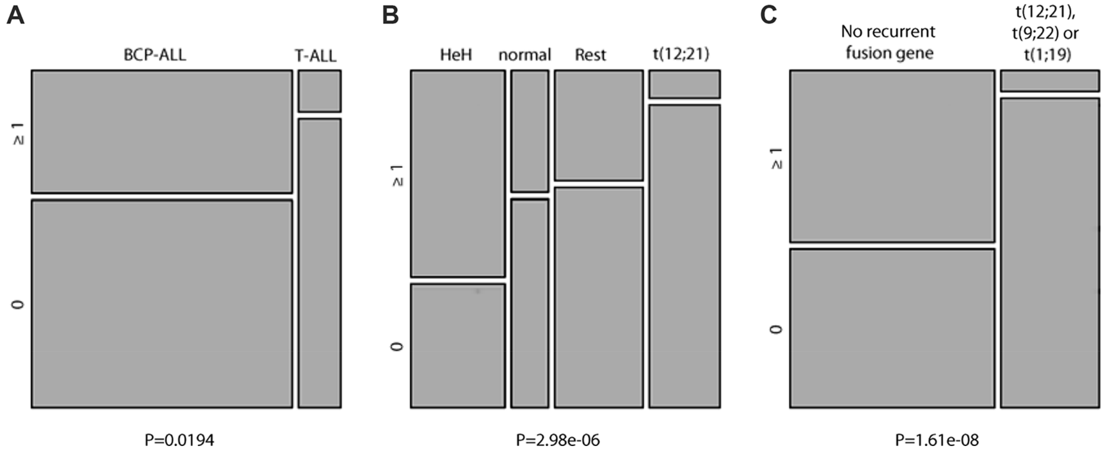

Supplementary Figure S10: Frequency of mutations in the Ras pathway in different patient subgroups. The panels show the proportion of patients with no and at least one non-silent mutation in the Ras pathway (*NRAS*, *KRAS*, *PTPN11* and *FLT3)* at diagnosis. All patients in the diagnostic cohort are shown. (**A**) As expected, BCP-ALL patients harbored more Ras mutations than T-ALL patients. (**B**) Significant differences in number of Ras mutations were observed between the major BCP-ALL subtypes, with the largest number of Ras mutations in HeH patients and the lowest number in t(12;21) patients. (**C**) The largest difference in number of Ras mutations was observed between BCP-ALL patients that carry any of the recurrent fusion genes *ETV6-RUNX1*, *BCR-ABL* or *TCF3-PBX1*, which harbored exceptionally few Ras mutations, and the remaining BCP-ALL patients.

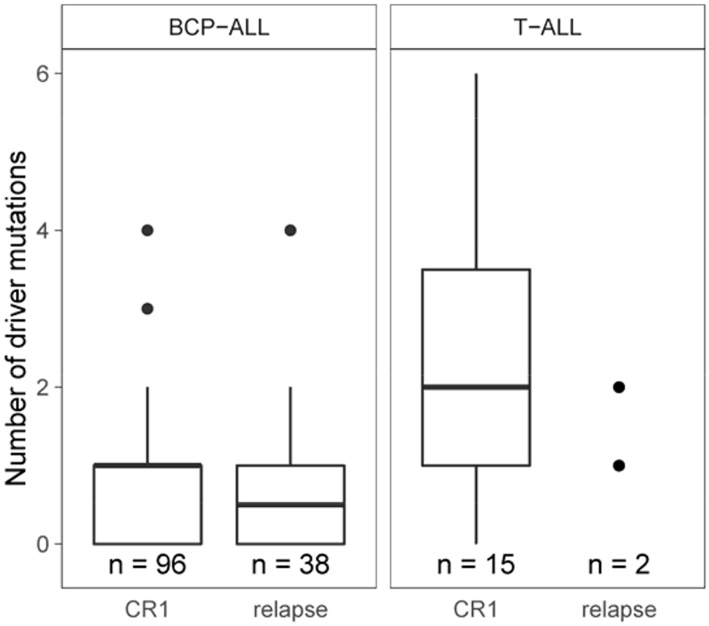

Supplementary Figure S11: Number of putative driver mutations in ALL patients with different clinical outcome. Boxplots showing the median number of non-silent mutations detected in 19 predicted driver genes in BCP-ALL and T-ALL patients with different clinical outcome. No significant difference in number of putative driver mutations was observed between patients with different clinical outcome. Ten BCP-ALL patients and three T-ALL patients with a shorter follow-up time than five years were excluded from the analysis. CR1, clinical remission 1.

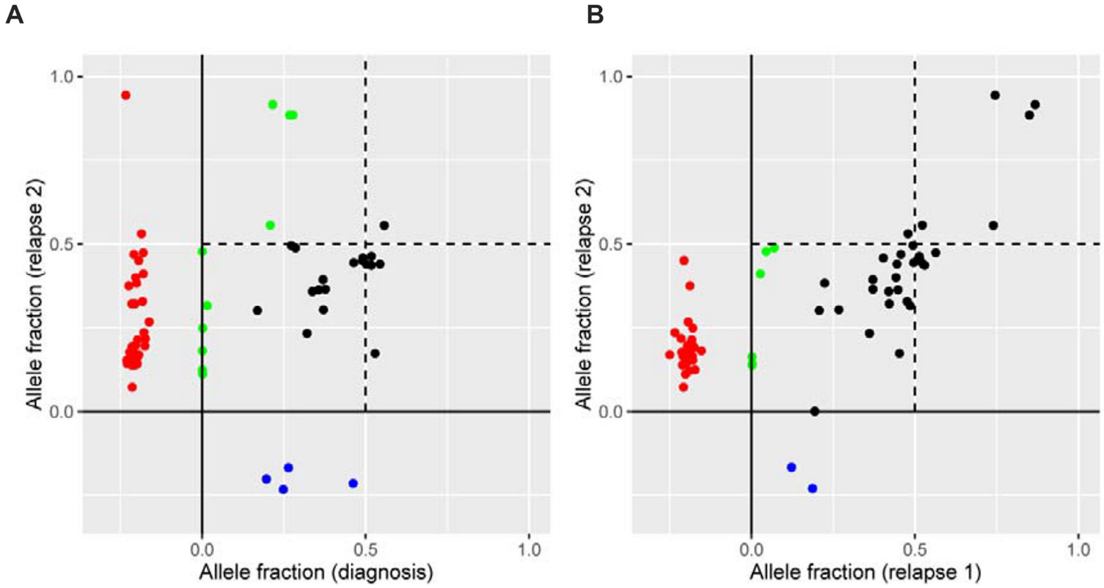

Supplementary Figure S12: Allele fractions of somatic mutations detected at relapse. Allele fractions of somatic mutations detected in the 19 patients from whom relapse samples were sequenced at (**A**) diagnosis and second relapse, and (**B**) first and second relapse. Mutations detected at both time points are shown in the large square, and those with indications of being part of expanding clones are highlighted in green. The rectangular areas at the bottom and the left show mutations only detected at the earlier time point (blue) and the later time point (red), respectively.

Supplementary Table S1: Genes included in the target capture experiment.
See Supplementary_Table_S1

Supplementary Table S2: Clinical characteristics of 172 ALL patients included in the diagnostic cohort. See Supplementary_Table_S2

Supplementary Table S3: Clinical characteristics of 19 ALL patients from whom relapse samples were sequenced. See Supplementary_Table_S3

Supplementary Table S4: Somatic SNVs and indels called in the diagnostic cohort.
See Supplementary_Table_S4

Supplementary Table S5: Non-silent somatic SNVs and indels detected in ATRX, SYNE1, the genes in the Notch signaling pathway, and genes putatively associated with relapse.
See Supplementary_Table_S5

Supplementary Table S6: Somatic SNVs and indels called at first relapse.
See Supplementary_Table_S6

Supplementary Table S7: Somatic SNVs and indels called at second relapse.
See Supplementary_Table_S7

Supplementary Table S8: Immunophenotypes and genetic subtypes of the ALL patients in the extension cohort

Immunophenotype	Genetic subtype[a]	No. of patients
T-ALL	T-ALL	16
BCP-ALL	HeH	44
BCP-ALL	Other	35
BCP-ALL	t(12;21)	29
BCP-ALL	Normal	18
BCP-ALL	dic(9;20)	8
BCP-ALL	No result	5
BCP-ALL	11q23/MLL	5
BCP-ALL	t(9;22)	3
BCP-ALL	t(1;19)	2
BCP-ALL	iAMP21	2
BCP-ALL	Susp HeH	1

[a]HeH, high hyperdiploidy (51-67 chromosomes); t(12;21), translocation between the chromosomes (12;21)(p13;q22)*ETV6-RUNX1*; t(9;22), translocation between the chromosomes (9;22)(q11;q34)*BCR-ABL1*; 11q23/MLL, translocation between *MLL* and various other genes; iAMP21, intrachromosomal amplification of chromosome 21; t(1;19), translocation between the chromosomes (1;19)(q23;p13)*TCF3-PBX1*; dic(9;20), dicentric chromosome (9;20)(p13;q11); Other, other clonal aberrations; Normal, no genetic aberrations detected and a normal karyotype observed in at least 5 of 25 metaphases; No result, no karyotype reported or the cytogenetic analysis failed; Susp HeH, suspected HeH.

REFERENCES

70. Liu X, Wang L, Zhao K, Thompson PR, Hwang Y, Marmorstein R, Cole PA. The structural basis of protein acetylation by the p300/CBP transcriptional coactivator. Nature. 2008; 451:846–850

71. Lauffer BE, Mintzer R, Fong R, Mukund S, Tam C, Zilberleyb I, Flicke B, Ritscher A, Fedorowicz G, Vallero R, Ortwine DF, Gunzner J, Modrusan Z, et al. Histone deacetylase (HDAC) inhibitor kinetic rate constants correlate with cellular histone acetylation but not transcription and cell viability. The Journal of biological chemistry. 2013; 288:26926–26943.
